# In Situ Observation of Shear-Induced Jamming Front Propagation during Low-Velocity Impact in Polypropylene Glycol/Fumed Silica Shear Thickening Fluids

**DOI:** 10.3390/polym14142768

**Published:** 2022-07-06

**Authors:** Anatoli Kurkin, Vitali Lipik, Xin Zhang, Alfred Tok

**Affiliations:** 1 School of Materials Science and Engineering, Nanyang Technological University, Singapore 639798, Singapore; kurk0001@e.ntu.edu.sg (A.K.); vitali@ntu.edu.sg (V.L.); 2 Department of Mechanics and Aerospace Engineering, Southern University of Science and Technology, Shenzhen 518055, China

**Keywords:** shear thickening fluid, impact behavior, jamming, discontinues shear thickening, impact absorption

## Abstract

Shear jamming, a relatively new type of phase transition from discontinuous shear thickening into a solid-like state driven by shear in dense suspensions, has been shown to originate from frictional interactions between particles. However, not all dense suspensions shear jam. Dense fumed silica colloidal systems have wide applications in the industry of smart materials from body armor to dynamic dampers due to extremely low bulk density and high colloid stability. In this paper, we provide new evidence of shear jamming in polypropylene glycol/fumed silica suspensions using optical in situ speed recording during low-velocity impact and explain how it contributes to impact absorption. Flow rheology confirmed the presence of discontinuous shear thickening at all studied concentrations. Calculations of the flow during impact reveal that front propagation speed is 3–5 times higher than the speed of the impactor rod, which rules out jamming by densification, showing that the cause of the drastic impact absorption is the shear jamming. The main impact absorption begins when the jamming front reaches the boundary, creating a solid-like plug under the rod that confronts its movement. These results provide important insights into the impact absorption mechanism in fumed silica suspensions with a focus on shear jamming.

## 1. Introduction

Shear thickening fluid (STF) is a type of colloidal suspension that possesses a non-Newtonian behavior under shear deformation. More specifically, it is a dilatant, where its shear viscosity increases beyond a critical shear rate. The shear thickening phenomenon exhibited is reversible, meaning the material returns to its initial liquid state upon removal of the applied stress [1]. The viscosity of STF can increase by a few orders in magnitude depending on particles fraction concentration, particles surface chemistry, particles aspect ratio, size, chemical structure, and molecular weight of the liquid medium [2,3,4,5,6]. Typical STF forms stable suspension due to steric, electrostatic, or Brownian interactions [7]. The mechanism of shear thickening was recently re-explained using the contact rheology model [8,9,10,11,12] after reconsidering the old explanation by the hydrodynamic model [7]. It was found that pressure created by hydrodynamic forces is not enough to overcome repulsion forces to form clusters. The contact rheology model suggests that the main contribution to the shear thickening is due to particle–particle interactions, not the particle–medium ones. After reconsideration, two types of shear thickening were introduced [1]: continuous shear thickening (CST), which is a weak type of shear thickening mainly driven by hydrodynamic forces, and discontinuous shear thickening (DST), which results in a discontinuous jump in shear stress beyond a critical shear rate due to contact forces between particles. A relatively new concept of jamming by shear (SJ) was introduced as an extreme behavior of DST suspensions below jamming particles fraction $\phi_{J}$ for a range of suspensions with sufficiently strong frictional interactions [12]. SJ is a reversible transition of unjammed dense suspension to a solid-like state with finite yield stress induced by shear [13,14,15].

Sliding and rolling friction between particles are among the main contributors to the DST and SJ [16]. Manipulation with the surface roughness [17], aspect ratio [18], adhesion [19], and chemistry [20] were shown to allow to tune the thickening, as well as to shift the onset. Surface chemistry (hydrogen bonds, in particular) was found to be essential for DST and SJ, where surface roughness cannot lead to DST alone [20,21].

Despite the variety of studies on a cornstarch water suspension, fumed silica was found to be a preferable choice for many applications due to its lower weight per unit volume. Fumed silica is produced from pyrolyzing tetrachlorosilanes in a hydrogen–oxygen flame, forming primary particles (5–30 nm) that cool to form silica aggregates (100–500 nm). This gives it a fluffy white appearance characterized by extremely low bulk density (20–50 g/L), high surface area (125–200 m^2^/g), and high aspect ratio [22]. The large surface area of fumed silica is dominated by the presence of silanol groups that are prone to form hydrogen bonds. In fumed silica/polypropylene glycol (PPG) colloidal suspensions, the terminal hydroxyl bonds, as well as internal oxygen atoms on the PPG chains, form hydrogen bonds with the abundant surface silanol groups on fumed silica. The interaction surface between PPG and fumed silica acts as a solvation layer that hinders the interaction between fumed silica particles, hence stabilizing the dispersion of fumed silica in the suspension [23]. In most studies on fumed silica STF rheology [2,12,23,24,25,26], little attention has been given to the nature of shear thickening (CST or DST). An attempt was made by Bourrianne et al. [21], where CST and DST transitions were found to occur starting from $\phi_{DST}≅6\%$ for hydrophilic fumed silica-based STF, which is considerably lower than for spherical particles. However, the stresses produced by steady-state rheology are very limited, which makes it hard to achieve and study SJ [27].

As was shown earlier [20], interparticle hydrogen bonding can be a source of strong frictional interaction, which may lead to SJ in dense suspensions. Since fumed silica surface is silanol rich with a high area, it suggests that it may possess shear jamming behavior at high concentrations/stresses. The first evidence of SJ in fumed silica suspension was provided by Naald et al. [6], where they observed it during impact using high-speed ultrasound imaging. They found that SJ was achieved only at the intermediate molecular weights of PEG due to the optimal thickness of the solvation layer, which allows the forming of frictional contact networks rather than lubricated contacts.

Unique dynamic properties of STF have found applications in the protective field, from sports protective equipment to flexible body armor [2]. However, the origin of impact absorption has been debated for many years, especially when it comes to jamming during compressive low-velocity impact. Two main mechanisms were proposed: jamming by densification [28,29] and by shear [30,31,32,33,34], where the second requires sufficiently strong frictional interactions between particles, such as via hydrogen bonding [20].

In this paper, we provide the evidence that shear-induced jamming transition is the main contributor to impact absorption in fumed silica STF. We utilize direct optical method to observe the jammed front formation and growth during impact. The big advantage of our STF is the total optical transparency due to the amorphous nature of fumed silica, which allowed us to record and track the displacements of dispersed dye particles during impact. DST was observed at all three weight fractions (15%, 20%, and 25%) during the steady-state rheological analysis, which is a prerequisite for SJ. Jamming front propagation speed was found to be 3–5 times higher than the speed of the impactor rod, which ruled out jamming by densification. These data were correlated with the results of the drop weight test; it was shown that the main impact absorption starts at the point when the jammed front reaches the bottom boundary. The thickness of the jammed front was directly correlated to peak forces, hence an impact absorption of STF. Therefore, this study provides useful insights for the fundamental research of shear thickening and jamming as well as for engineering research to design and tailor STF for particular requirements.

## 2. Materials and Methods

Polypropylene glycol (PPG) 400 and hydrophilic fumed silica with 0.2–0.3 µm average particle size and surface area of 200 m^2^/g (S5505) were purchased from Sigma-Aldrich. STF with 15%, 20%, and 25% weight fraction of fumed silica were prepared by first weighing out PPG400 and, respectively, the amount of fumed silica required. A beaker consisting of PPG400 was mixed using a high shear mixer (Silverson L4RT), while small volumes of fumed silica were added gradually to the mixture. The mixture was stirred until all fumed silica was evenly integrated. The beaker with the mixture was placed in a sonicator bath until all air bubbles disappeared. The resultant mixture was a clear colloidal suspension, with varying viscosities depending on the weight fraction of fumed silica used. A fumed silica true density value of 2.3187±0.0012 g/cm^3^ was used from the literature [35], where it was obtained for hydrophilic fumed silica with the same surface area (200 m^2^/g) to calculate volume fractions from the weight fractions. Based on the calculations, the weight-to-volume ratio is 2.086. Hence, $\phi_{15\%w}≅7.2\%$, $\phi_{20\%w}≅9.6\%$, $\phi_{15\%w}≅12.0\%$. However, weight fractions were used along with the manuscript in view of the greater prevalence of weight fraction values in the literature on fumed silica suspensions. The densities of STF were measured using volumetric flask/calipers and weights. The values were as follows: 1.06 g/cm^3^, 1.10 g/cm^3^, and 1.14 g/cm^3^ for STF 15%, 20%, and 25%, respectively.

The flow rheological properties of STF are tested using a rheometer (Anton Paar, MCR501) with 25 mm diameter parallel plates (PP-25) and a gap of 0.5 mm to ensure adequate filling of the STF over the testing disk. All tests were conducted at 25 °C. A coin-sized amount of STF was poured onto the Peltier plate, followed by the lowering of the parallel plate to the set gap width. The excess STF leakages at the edge of the parallel plate were trimmed using a task wiper, ensuring that the entire surface area of the top plate was in contact with the STF.

**Figure 1:**
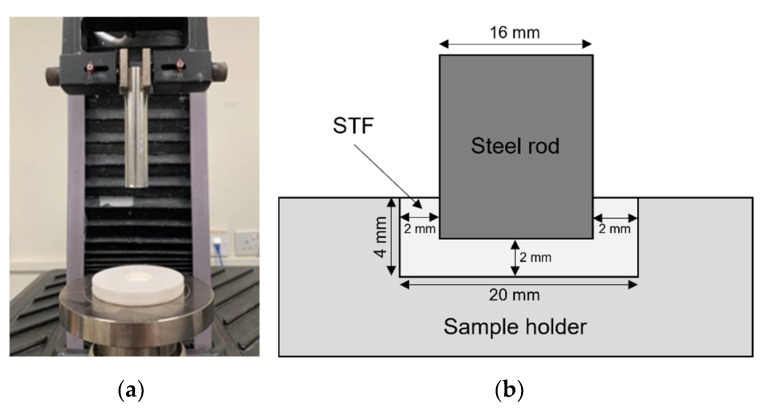
Custom setup for pull-up tests: (**a**) Photo of the setup with a pneumatic grip holding the cylindrical rod at the top and the sample holder attached at the bottom; (**b**) Schematic illustration of the starting position during the pull-up test with all dimensions shown.

A pull-up test was designed to achieve higher shear stresses without fluid slippage. A mechanical tester, MTS Criterion Model 42, was used to measure the normal force of the STF when the submerged rod is retracted upwards. The equipment set-up is shown in Figure 1a, where a top pneumatic grip is attached onto the load cell and used to clamp the steel rod in place. A 3D-printed polylactic acid (PLA) sample holder with a storage diameter of 20 mm and a depth of 4 mm was also attached firmly onto the bottom steel plate using highly adhesive double-sided tape.

The schematic diagram below (Figure 1b) shows the starting position for the tests. The steel rod had a diameter of 16 mm to ensure that a fixed distance of 2 mm was observed between the rod and the bottom and sides of the sample holder. The sample holder was approximately ¾ filled with STF before the rod was lowered into the sample. After that, any excessive fluid was removed until a horizontal level was achieved. The rod was lifted at a speed ranging from 0.1 mm/s to 30 mm/s and the normal force experienced by the rod was recorded.

**Figure 2:**
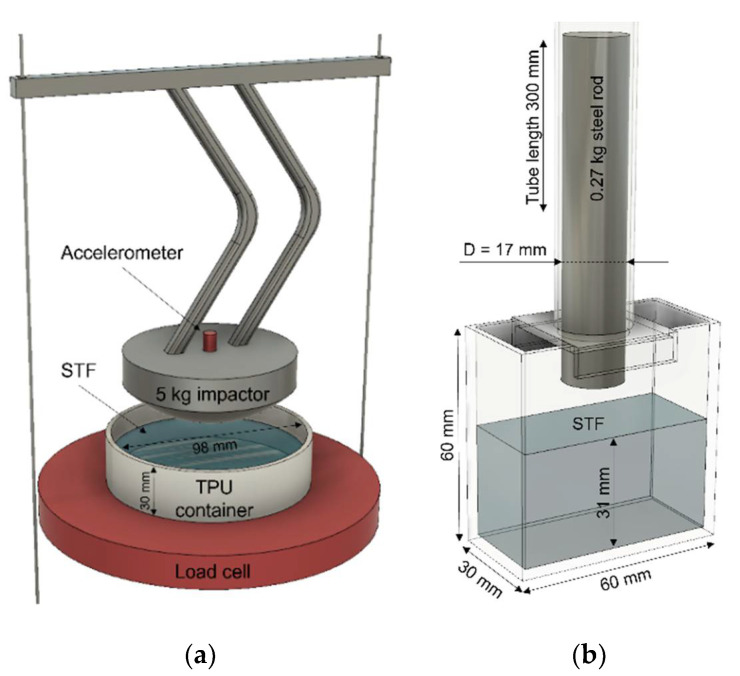
Illustrations of low-velocity impact setups. (**a**) The drop weight impact setup with a custom 3d printed TPU container. All samples were filled to the 13 mm thickness level from the bottom. Height from the surface of STF to the tip of the hemispherical impactor was a controlled parameter. (**b**) The acrylic glass setup that was used together with a speed camera for the flow field analysis during impact. All samples were subjected to the impact from the same height (327 mm).

Drop weight impact tests on bulk samples were carried out on the CADEX twin wire flying arm machine. The illustration of the setup with all dimensions is shown in Figure 2a. The hemispherical impactor was attached to the flying arm with a total drop weight of 5 kg. An accelerometer and 44 kN load cell were used for deacceleration and ground reaction force registration. Flexible 3D-printed thermoplastic urethane (TPU) carrier (with 98 mm in diameter and 30 mm in height) was used as an STF holder. The setup was designed the way that the impactor tip does not touch the walls of the soft container. All STF samples were filled to the same level in a container with a total thickness of 13 mm. At least 10 times impact tests were repeated for each sample at each height to make sure that the results are reproducible and are within 10% error. Then, all results were averaged. Displacement was calculated from the integration of the velocity–time plot, which in turn was obtained by integrating the force–time plot divided by the impactor weight. Impact energy was calculated from the simple equation E=m·g·h, where the impactor mass m was 5 kg and constant, gravitational acceleration g was used as 9.8 m/s, and constant and impactor height h was a variable.

Another impact test was designed to visualize and analyze the displacement of fluid during the impact (Figure 2b). Sheets of 2 mm thick clear acrylic glass were used for the setup, which allowed to optically inspect the STF during the impact. All dimensions are present in Figure 2b; the geometries of the container and tube were designed to securely hold the tube during the impact. The diameter of the rod (17 mm) and the weight (0.27 kg) were chosen to match the stress level of the hemispherical impact. STF was filled to the same level (31 mm) and a small concentration of black SiO_2_ microparticles (<0.1% by weight) were added to suspensions as a dye to track displacement of different parts of the fluid. A speed camera Phantom Miro M120 was used at 7300 fps resolution to capture the flow dynamics. MatchID digital image correlation (DIC) software was used for the flow-field displacement calculations of STF during impact. The vertical component of normal strain $\varepsilon_{x}$ was calculated for each point of strain window per frame. Then, points with the same strain value were colored from red for the biggest value to yellow for the lowest. The maximum thickness of the third strain isoline (dark orange) in a vertical direction was used as a jammed front thickness value.

## 3. Results and Discussion

### 3.1. Rheological Analysis of STF with Different Particles Concentrations

**Figure 3:**
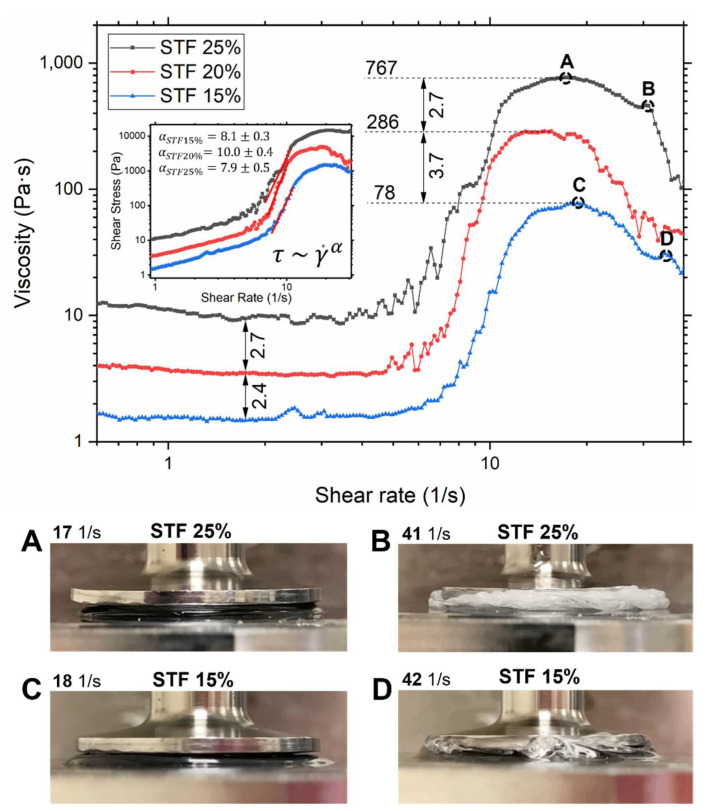
Flow rheological analysis of STF at three weight fractions. Viscosity as a function of shear rate in a parallel plates geometry with a 0.5 mm gap. Numbers on the arrows represent ratios of viscosities at different stages, the first two values at the bottom during the Newtonian stage, values on top ratios at the maximum viscosities. An additional graph inside the main is a shear stress-rate plot, which was used for power-law fitting to establish the regime of shear thickening (DST or CST). (**A**,**C**) Photos of STF 25% and 15% during flow tests at the maximum viscosity when fluid starts to slip off (17 1/s and 18 1/s, respectively), (**B**) when STF 25% becomes cloudy (41 1/s); (**D**) in contrast to STF 25%, STF 15% did not demonstrate any change in optical properties (at 42 1/s).

Figure 3 shows the rheological flow profiles of STF with three different concentrations of fumed silica (15, 20, and 25% by weight). Each graph can be split into three different zones: shear thinning, Newtonian, and shear thickening zones. The first zone is a shear thinning zone where the viscosity decreases with shear rate. The behavior in this zone is characterized by the net of three contributions: a Newtonian portion of constant viscous stress, an entropic portion of randomly colliding particles under thermal motion [36,37], and alignment of polymer chains due to polymer nature of the liquid medium. All STF demonstrated very mild shear thinning due to the low molecular weight of PPG, hence the higher conformational freedom of chains as well as the insignificant contribution from the entropic interactions. Next is the Newtonian zone with an almost invisible transition. The constant value of the viscosity in this zone represents the laminar properties of the fluid without effect from the particle’s interactions [36]. In order to assess the type of shear thickening (CST or DST) present in our STF, we fitted shear stress-rate curves by power-law $\tau \sim {\dot{\gamma }}^{\alpha }$ in the shear thickening zone (Figure 3). According to Jaeger et al. [1], 1<α≤2 represents CST, where α>2 represents DST. In our case, α > 7 for all three particles concentrations ($\alpha_{STF15\%}=8.1\pm 0.3;\;\alpha_{STF20\%}=10.0\pm 0.4;\;\alpha_{STF25\%}=7.9\pm 0.5$), which clearly indicates the presence of DST. Therefore, we can conclude that frictional interactions between particles are strong enough to trigger a discontinuous jump in shear stress. The nature of these strong frictional interactions is the cumulative of the contributions from hydrogen bonding between the silanol-rich surfaces and the highly anisotropic shape of fumed silica [6]. According to another study [21], the onset volume fraction of DST $\phi_{DST}$ for hydrophilic fumed silica suspension was found to be 6%, which is below our lowest weight fraction (15% by weight corresponds to 7.2% by volume). Hence, this study supports our results that DST is present in all selected weight fractions.

In the literature on STF, two parameters of flow rheology were extensively used and studied: the critical shear rate and maximum viscosity. It is apparent that the critical shear rate decreases as the particle concentration increase, as shown by the regions before an increase in viscosity. This supports the hydrodynamic theory [38], which comes into play at the onset of shear thickening that shows that, at higher particle concentrations, stronger hydrodynamic forces are present due to shorter interparticle distance. Thus, a lower shear rate is required to overcome the repulsive forces, and shear thickening occurs at the lower shear rate at a higher particle concentration. However, the difference between the values is insignificant. The points of maximum viscosity shown in Figure 3 increase with increasing particle concentration as frictional contact forces dominate and particle mobility is reduced. An interesting feature of maximum values is that their ratios follow the same trend as values in the Newtonian region and are roughly equal.

However, the portrayed maximum viscosity is irrelevant in depicting the true maximum viscosity of the STF as slipping occurs past a certain shear rate. Slipping occurs commonly in viscous suspensions due to the large velocity gradient between the plates and the fluid. During slipping, the fluid is no longer held between the parallel plates and the recorded viscosity will be much lower than the true viscosity [39]. Slipping decreases the surface tension between the fluid and plates. This fact combined with high centrifugal force at higher shear rates lead to the loss of fluid between the plates and, therefore a drop in viscosity. Figure 3A,C shows the STF samples confined between the parallel plates at maximum viscosity; it can be observed that the STF samples started to flow out of the parallel plate boundary and onto the Peltier plate where their viscosities can no longer be recorded. It can be seen more clearly at higher shear rates (B and D in Figure 3) that fluids slip and flow out. An interesting feature was noticed at shear rates beyond the maximum viscosity for STF 25%: the optical properties changed from transparent to translucent white. That happens because of the Tyndall effect. After a certain shear rate, particles start to agglomerate into clusters, where these clusters are large enough to start scattering visible light and make the colloidal suspension appear cloudy. The cluster formation is induced by hydrodynamic lubrication forces as was explicitly shown using fast confocal microscopy combined with simulations force measurements during rheological experiments [37]. On the contrary, STF 15% did not demonstrate any change in optical properties. Therefore, shear stress was not large enough to reach the certain size of hydroclusters for the scattering of visible light due to the lower concentration of fumed silica.

As aforementioned, we can conclude that the data recorded after slipping do not provide relevant information about the true viscosity of the STF, rendering the data after the fall in viscosity irrelevant. Shear stresses, which are required to achieve a shear jammed state, cannot be reached with a conventional rheometer due to failure modes [27].

In order to overcome this challenge, a custom extensional test was designed as it allows us to achieve higher shear stresses without any slippage of the fluid. Another advantage of extensional rheology is that it was found to be more relevant to the impact mechanics. It was shown earlier that extension of STF at rates higher than onset rates of jamming transition resembles mechanics of structural changes under impact [27]. It particularly supports the concept of jamming by shear where still and moving portions of fluid generate strong localized shear that triggers the formation of frictional networks of particles [30,40]. A modified extensional test was carried out in accordance with another study [20] with the sample holder and the rod dimensions doubled. Peak forces were recorded at each extension rate.

**Figure 4:**
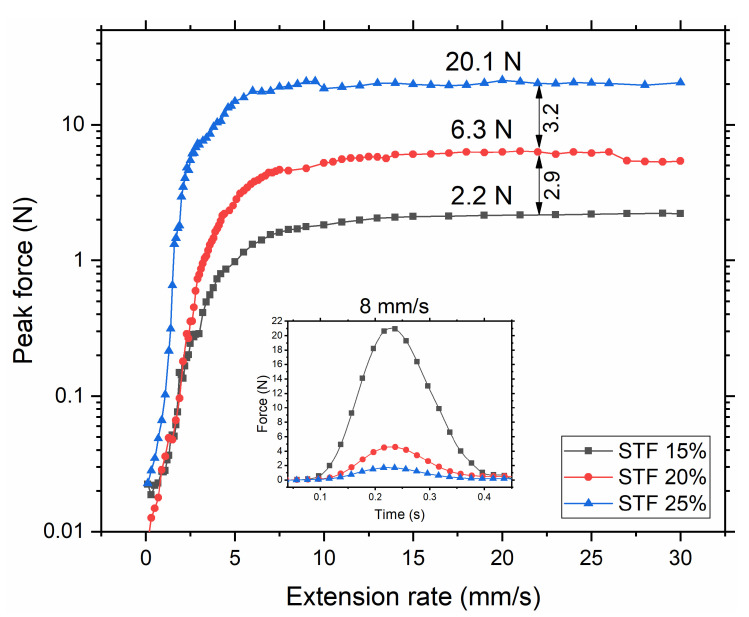
Extensional “pull-up” test. Peak forces at each individual extensional rate test are plotted against the rate, and peak forces are plotted in a log scale. Normal force profile at 8 mm/s is shown in the middle of the figure; at this rate, peak force values reach the plateau.

As seen in Figure 4, the general trend across all samples is an increase in peak force from 0.1 mm/s to 8 mm/s, followed by a plateau. The rate of increase in peak force, given by the gradient of the upward curve, is drastically higher with higher weight concentrations of fumed silica considering the peak force was plotted on a log scale. The ratios of plateau values resemble the ratios between maximum viscosities in flow tests (Figure 3). Since at higher shear stresses the same trend is observed, the maximum flow viscosities can be used for the further interpretation of the impact tests.

All curves reach a plateau around 8 mm/s. This value of peak force represents the maximum DST/SJ exhibited by the STF sample during the extensional tests. Comparing the peak force at 8 mm/s, the STF 25% sample shows an 11.7 times higher peak force than that of the 15% sample. In James et al.’s experiment [20], the presence of shear jamming is determined by a significant increase in normal force recorded at 8 mm/s. The peak force value of 12.0 N in PMMA/ITA suspension was shown to originate from SJ rather than just from shear thickening, whereas a non-SJ glass beads suspension demonstrated only 1 N of normal peak force at the same extension rate. Drawing parallels with our results (20.1 N for STF 25% at 8 mm/s), it can be concluded that the high normal force in STF 25% is attributed to the presence of shear jamming. It is also worth noting that a pulling force of 20.1 N for 25 wt% STF corresponds to stress exceeding 65 kPa, which is noticeably larger than that achieved during flow tests. This stress far exceeds stresses from the lubrication model or stresses that can be achieved with capillary forces [1,27,41]. Therefore, such high stresses originate from the frictional interaction between the particles. Sterically dispersed particles need to overcome repulsive forces to come into contact with each other, which causes a drastic increase in peak force.

### 3.2. Low-Velocity Impact Behavior of STF

**Figure 5:**
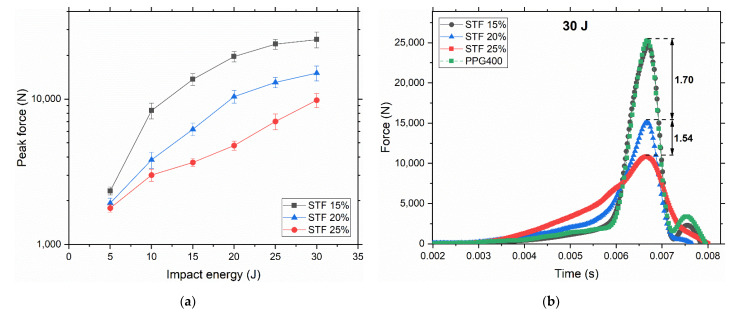
Results from the drop weight test with the hemispherical impactor. (**a**) Peak forces at six different energies with error bars. Percentages represent the weight fraction of fumed silica in STF. Each value is the average of ten tests with an error not exceeding 10%. (**b**) Normal force registered with the load cell as a function of time during 30 J impact. These curves are also the average over ten tests.

Drop weight impact test was used to analyze the impact response of STF in the low velocities range. The 13 mm thick layer of STF was subjected to six different impact energies based on the height of a 5 kg hemispherical impactor. The peak forces of three STF with different fumed silica concentrations are present in Figure 5a. The peak forces were plotted on a logarithmic scale to correlate with rheological data. At 5 J, all STFs demonstrated sufficiently low and close values of peak forces, suggesting that the impact was within their capacity. After 5 J, the curves start to move away from each other. The peak force of STF 15% increased over three times at 10 J with a relatively consistent growth after. The shape of the curve, however, suggests that there is a plateau to which this curve tends. STF 20% demonstrated an almost linear growth until 20 J with the same trend as for STF 15%, where peaks forces increase less and may eventually reach the plateau. This suggests that, after 20 J, STF reaches the maximum capacity to bear the impact with the aid of DST/SJ. On the contrary, STF 25% showed an almost linear increase in the whole range of energies, suggesting that the maximum capacity of impact absorption has not been reached yet. This hypothesis can be proved if we analyze individual impact profiles at 30 J (Figure 5b). STF 25% shows an almost linear increase in force with time where STF 20% and especially STF 15% demonstrate exponential increase with a sharp peak force. Judging by the force distribution over time, STF 25% starts to absorb energy first (the slope of the force curve at the start of the impact is the highest). Therefore, impact absorption is “activated” the earliest in STF 25%. It is aligned with the fact that particles at higher concertation come into contact more easily, triggering DST/SJ the earliest, which provides a massive impact dissipation (via friction between particles). However, additional tests will be conducted to understand the nature of impact dissipation. As it was suggested by the referee, the liquid medium alone (PPG400) was subjected to an impact of 30 J to evaluate the impact mitigation performance of PPG400 on its own. Surprisingly, we found that STF 15% and PPG400 curves coincide, demonstrating the same peak forces and the same distributions. As it was said earlier, the impact absorption capacity of STF 15% is limited to 5 J since it demonstrated a jump in a peak force at 10 J. Therefore, since the impact bearing capacity was far exceeded at 30 J for STF 15%, DST/SJ and/or hydrodynamic forces do not contribute enough to impact absorption to be noticed.

To explain this phenomenon, we conducted an impact test in a transparent container with a speed camera. The great advantage of this type of STF is that, due to the amorphous nature of fumed silica, suspensions remain optically transparent at all concentrations. This allowed us to optically observe the flow of fluid during the impact. A small concentration of SiO_2_ particles was added to suspensions to track the displacements of different parts of the fluid more clearly.

**Figure 6:**
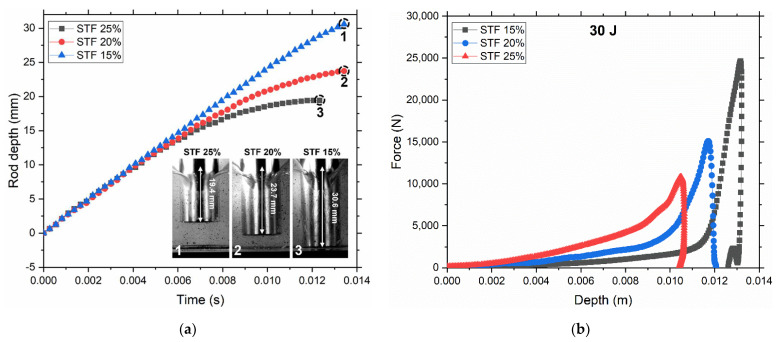
Change in impactor depth for two types of impact tests and the resemblance between them. (**a**) Rod depth against time during speed camera impact test. The speed of the rod before the impact $\nu_{0}=2.4$ m/s. Photographs from the speed camera when the rod is completely stopped (19.4 mm for STF 25%, 23.7 mm for STF 20%, and the rod hit the bottom for STF 15% at 30.6 mm depth). Recording of whole tests is shown in Supplementary Video S1. (**b**) Calculated force-displacement (depth) profiles for drop weight test at 30 J. Displacement was calculated from the integration of the velocity-time plot, which in turn was obtained by integrating the force-time plot divided by the impactor weight.

First, it was found that, in the case of STF 25%, the rod started slowing down first, followed by STF 20%, and STF 15% started to slow down only near the bottom boundary (Figure 6a). The penetration depth of the rod linearly decreases with concentration. STF 25% demonstrated the best stopping efficiency with 19.4 mm depth, followed by 23.7 mm for STF 20%, and STF 15% gently hit the bottom at 30.6 mm depth. The speed of the rod before the impact $\nu_{0}$ is 2.4 m/s. The same behavior was demonstrated during the drop weigh test (Figure 6b) where the penetration depth of the impactor gradually increased from STF 25% to hit the bottom for STF 15%. Another resemblance between these two experiments is that the normal force starts to grow the earliest for STF 25% due to the formation of the jamming front, which slows down and eventually stops the rod.

**Figure 7:**
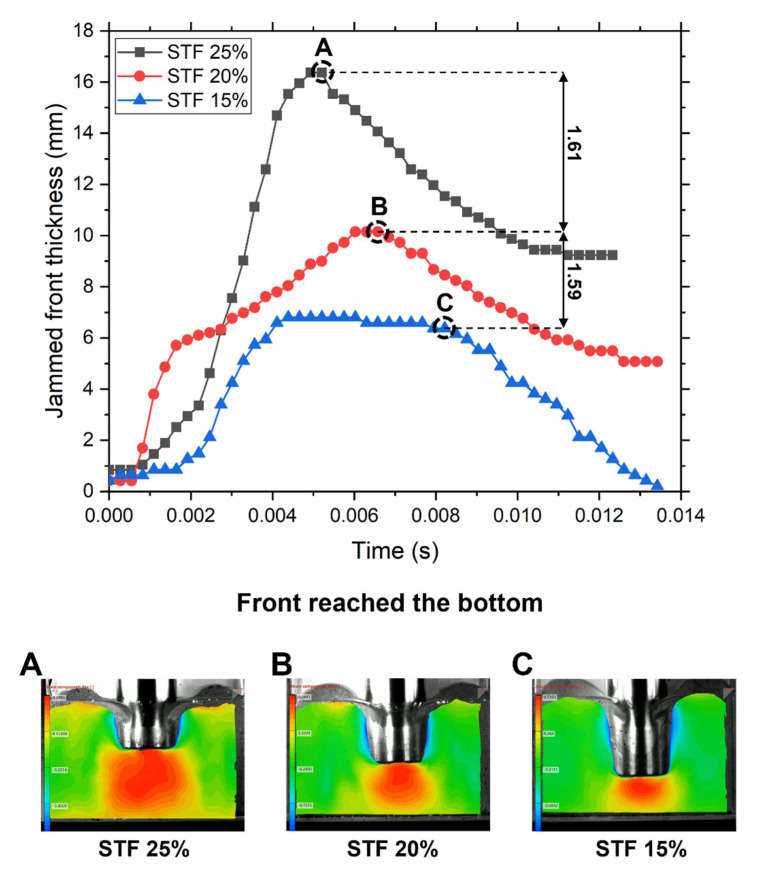
Jamming front propagation during impact. Calculated jamming front thickness during the impact of STF for three particles weight fractions. Front thickness was obtained measuring the maximum thickness of the third strain isoline (dark orange) in a vertical direction from the rod bottom surface. (**A**–**C**) Color-graded strain fields were calculated using DIC software. Red represents the highest displacement value, where yellow is the lowest and the green color represents no displacement. The red zone is the jamming front propagating towards the bottom at a speed faster than the speed of the rod. Captured pictures are front dimensions at the moment of reaching the bottom.

The next step was to analyze the displacement of particles in suspensions during the impact. It was possible to calculate the solidification front dimensions during impact using DIC software. Jamming front thickness was plotted against the time for three concentrations (Figure 7). The complete videos of solid front formation can be found in Supplementary Video S1.

Three distinctive phases were observed during the impact. The first phase is the growth of the jamming front followed by the second one, which is the propagation of the front. However, in the case of STF 25%, the first and second phases were combined into one. The front grew consistently all the way to the very bottom. STF 20% and 15% demonstrated propagation of the formed front to the bottom. However, the front of STF 20% kept growing in this phase whereas STF 15% did not. The third one takes place after the front reaches the boundary. The jammed layer is pushed against the boundary with the diffusion of particles to the sides due to compression. STF 25% solid front reached the boundary first followed by STF 20% and then STF 15%. We calculated jamming front propagation speed $\nu_{f}$ at the first stage of the impact, and it was found that velocities at all concentrations far exceed $\nu_{0}$. For STF 25%, $\nu_{f}=12.3$ m/s, which is five times faster than $\nu_{0}$; for STF 20%, $\nu_{f}=10$ m/s, which is four times faster than $\nu_{0}$; and for STF 15%, $\nu_{f}=6.5\;$ m/s, which is almost three times faster than $\nu_{0}$. The fact that the front propagation speed is much faster than the rod speed together with the extensional test results proves that the jamming is induced by shear, not by densification [27,31]. It should be noted that SJ was observed at all three concentrations at the impact stresses, but only STF 25% could enter the SJ regime during extension tests due to much lower stresses.

In the earlier investigations of the STF under impact [31], it was shown that normal force starts to grow drastically when the jamming front reaches the boundary. In our case, the front of STF 25% reaches the boundary first creating strong resistance for the rod, which leads to a drastic increase above the normal force. This can be confirmed by analyzing the impact profile in Figure 5b, and it was obvious that normal force starts to grow much faster (the slope value of STF 25% at the beginning of impact is two times higher than for STF 20% and three times higher than for STF 15%) than in the cases of STF 20% and 15%. The front of STF 20% reaches the boundary later with a much thinner layer; therefore, it generates less resistance for the rod, and a significant part of the impact is transferred to the load cell. The front of STF 15% reaches the boundary the latest, with the thinnest front layer providing insufficient resistance to effectively absorb the impact; this leads to a slow peak rise of a normal force and a high value of the peak force. The visualization of the front at the moment of reaching the boundary is present in Figure 7A–C. By analyzing front thicknesses at this moment, we can directly correlate them to peak forces generated during the drop weight test (Figure 5b). The ratio of front thicknesses between STF 15% and 20% is 1.59, where 1.54 is the ratio between their peak forces; the ratio of front thicknesses between STF 20% and 25% is 1.61, where 1.70 is the ratio between their peak forces. Therefore, such proximity of values supports the notion that the main portion of impact is absorbed by the SJ solid plug beneath the rod pushing against the direction of the impact [28,31]. A much larger and more uniformly distributed deformation zone (in red color) can be generated by the impact rod found in the suspension with higher concentration of particles, which indicates a stronger shear jamming and more stable stress wave equilibrium in the suspension with a higher particle concentration.

## 4. Conclusions

Putting it all together, we confirmed the presence of DST at all weight fractions (15, 20, and 25%) using flow rheology. However, it was impossible to achieve jamming transition due to failure modes. Extensional rheology helped to reach higher stresses without fluid slippage. STF 25% demonstrated similar peak forces to another study where they correlated it with the presence of SJ [20]. Low-velocity impact tests were conducted to study the mechanism of impact absorption and how SJ contributes. Owing to the optical transparency of fumed silica suspension, we directly observed the formation of the jamming front during impact. The speed of front propagation was calculated to be 3–5 times (3× for STF 15%, 4× for STF 20%, and 5× for STF 25%) higher than the impactor speed, which rules out jamming by densification. Therefore, it was induced by shear. Front propagation data were coupled with drop weight impact test results, suggesting that the main portion of impact was absorbed once the SJ front reaches the bottom boundary. The efficiency of impact absorption was found to depend on fumed silica fraction through the thickness of the front between the rod and the bottom. The ratios of front thicknesses are almost equal to the peak force ratios, which supports this hypothesis. Therefore, the high impact absorption of STF 25% over lower fractions was attributed to the formation of the SJ front growing much faster than the speed of the impactor to the boundary. This front reaches the boundary the earliest for STF 25% with the thickest jammed plug, which helps to mitigate the impact most efficiently. The penetration depth of the rod reflected the impact absorption characteristics of our STF, where STF 25% demonstrated the lowest depth, followed by STF 20%, and finally STF 15%; however, STF 15% hit the boundary. This study provides new insights on a mechanism of energy absorption in PPG/fumed silica STF with direct observation of the jamming front formation during low-velocity impact. Interestingly, fumed silica suspension demonstrated SJ at $\phi \ll \phi_{J}$.

## Data Availability

Data are available upon request via e-mail miytok@ntu.edu.sg.

## Supplementary Materials

The following supporting information can be downloaded at: https://www.mdpi.com/article/10.3390/polym14142768/s1, Video S1: Propagation of the jamming front.
